# Neuroprotective Effects of *Cornus officinalis* on Stress-Induced Hippocampal Deficits in Rats and H_2_O_2_-Induced Neurotoxicity in SH-SY5Y Neuroblastoma Cells

**DOI:** 10.3390/antiox9010027

**Published:** 2019-12-26

**Authors:** Weishun Tian, Jing Zhao, Jeong-Ho Lee, Md Rashedunnabi Akanda, Jeong-Hwi Cho, Sang-Ki Kim, Yu-Jin Choi, Byung-Yong Park

**Affiliations:** 1 College of Veterinary Medicine and Bio-safety Research Institute, Jeonbuk National University, Iksan 54596, Korea; tianws0502@126.com (W.T.); zhaojing19901230@126.com (J.Z.); uribugi@naver.com (J.-H.C.); 2 Sunchang Research Institute of Health and Longevity, 427-128 Indok-ro, Ingye-myeon, Sunchang-gun, Jeollabuk-do 56015, Korea; wooju0717@hanmail.net; 3 Department of Pharmacology and Toxicology, Sylhet Agricultural University, Sylhet 3100, Bangladesh; akandamr.dph@sau.ac.bd; 4 Imsil Cheese Livestock Cooperative Association, 275 Galma-ri, Imsil-eup, Imsil-gun, Jeollabuk-do 55924, Korea; korwor@nonghyup.com; 5 Imsil Cheese & Food Research Institute, 50 Doin 2-gil, Seongsu-myeon, Imsil-gun, Jeollabuk-do 55918, Korea; samdc@nate.com

**Keywords:** *Cornus officinalis*, immobilization stress, H_2_O_2_, oxidative stress, MAPK pathway

## Abstract

Oxidative stress plays a vital role in neurodegenerative diseases. *Cornus officinalis* (CC) has a wide range of pharmacological activities (e.g., antioxidant, neuroprotective, and anti-inflammatory). The present study was undertaken to elucidate the neuroprotective mechanism of CC and fermented CC (FCC) on stress and H_2_O_2_-induced oxidative stress damage in rats and SH-SY5Y cells. A dose of 100 mg/kg CC or FCC was orally administered to rats 1 h prior to immobilization 2 h per day for 14 days. CC, especially FCC administration decreased immobility time in forced swim test (FST), effectively alleviated the oxidative stress, and remarkably decreased corticosterone, β-endorphin and increased serotonin levels, respectively. In cells, CC and FCC significantly inhibited reactive oxygen species (ROS) generation, lactate dehydrogenase (LDH) release and significantly increased the genes expression of antioxidant and neuronal markers, such as superoxide dismutase (SOD), catalase (CAT), and brain-derived neurotrophic factor (BDNF). Moreover, the pro-apoptotic factor Bax and anti-apoptotic factor Bcl-2 (Bax/Bcl-2) ratio was regulated by CC and FCC pretreatment. Both in rats and cells, CC and FCC downregulated mitogen-activated protein kinase (MAPK) phosphorylation. Taken together, these results demonstrated that CC and particularly FCC ameliorated oxidative stress and may be used on the neuroprotection.

## 1. Introduction

Stress is a feeling of emotional or physical tension. Long-term exposure to stress dampens learning and memory, and causes a number of diseases, including depression, insomnia, anxiety, and post-traumatic stress disorder [1]. Immobilization stress is widely used to induce depressive-like and anxiety-like behaviors, and hippocampal neuronal damage in rodents [2,3]. The hippocampus is one of the most stress-prone targets because it expresses many glucocorticoid and mineralocorticoid receptors [4,5]. Stress alters hippocampal neuronal activity and synaptic plasticity, increases hippocampal glucocorticoid receptor activation, and reduces neuronal cell survival and neurogenesis [6,7,8].

Stress often encourages consumption to maintain normal body functions. The excessive production of free radicals causes oxidative damage to the brain [9,10]. Oxidative stress is induced by an imbalance between the generation of reactive oxygen species (ROS) and the ability of biological systems to detoxify active intermediates. The brain is one of the organs that is particularly susceptible to ROS because it requires much oxygen supply and is rich in peroxide-sensitive lipids. Among the various ROS, H_2_O_2_ is a major factor in the production of oxidative stress. Excessive ROS levels increase cellular oxidative stress, resulting in inactive enzymes, proteins oxidation, DNA damage, lipid peroxidation, and denaturation of protein, disrupting cell function and integrity. Neural cells exposed to H_2_O_2_ may undergo apoptotic-like delayed death and necrosis. Previous studies have verified that oxidative stress plays an important role in neurodegenerative diseases such as depressive-like behavior, memory impairment, anxiety, Parkinson’s disease, and Alzheimer’s disease [11,12]. There is also evidence that antioxidants can attenuate oxidative stress-induced neuronal cell damage [13].

*Cornus officinalis* (CC), also known as the “*Cornelian cherry*”, is a traditional medicine with strong antioxidant [14] and anti-inflammatory effects [15]. CC extract and active components such as vitamins, fatty acids, amino acids, and bioactive compounds, including loganin [16], loganic acid [17], cornin [18], sweroside [19], and cornuside [20] have numerous biological activities, including antioxidant, and neuroprotective activities. Among them, loganin plays a vital role in the formation sequences of highly oxidized glucosides of iridoids and the biosynthesis of secoiridoids glucosides and indole alkaloids [21]. CC contains a large number of phenolics and flavonoids. Furthermore, fermentation technology can reduce toxicity and improve absorptive capacity by altering the molecular structure of organic materials, producing bioactive compounds, and promoting health. Fermentation microbes such as *Lactobacillus rhamnosus*, *Enterococcus faecium*, and *Lactobacillus acidophilus* have been testified as sources of polyphenols [22], which can improve nutritional quality [23], and enhance various bioactivities including antioxidant and immune stimulation [24]. Although there have been several studies on the properties of CC, there is still unclear the impact of CC and fermented CC (FCC) on fundamental cellular pathways to understand its beneficial effects in a neuronal sense. The present study was designed to investigate the effect of CC and FCC on stress-induced hippocampal deficits in rats and H_2_O_2_-induced neurotoxicity in SH-SY5Y human neuroblastoma cells.

## 2. Materials and Methods

### 2.1. Preparation of CC and FCC Extract

*Cornus officinalis* (CC) was provided by Omnihub Company (Daegu, South Korea) in October 2015 and verified by professor Jong-Sik Jin from Jeonbuk National University, Jeonju, South Korea. CC was shade-dried at room temperature and shattered using a freeze dryer (FDA5508, ilShinbiobase. Co., LTD, Dongducheon, South Korea). CC and FCC extracts were prepared according to the published method [25]. Briefly, 20 g CC power was extracted in 400 mL water and stirred at 100 °C for 9 h. The extract was filtered and the filtrate was concentrated on a rotary evaporator, lyophilized, and sterilized at 121 °C for 15 min. For FCC extract preparation, *Lactobacillus rhamnosus*, *Enterococcus faecium*, and *Lactobacillus acidophilus* were mixed (1:1:1, 1.0 × 10^6^ U/mL) and added to CC extract. CC extract was fermented at 37 °C for 48 h, and the supernatant was collected after centrifugation (10,000× *g*, 5 min, 4 °C) and sterilized for 15 min at 121 °C. The dried extract was kept at −20 °C.

### 2.2. Loganin, Total Phenolic, and Flavonoid Contents Detection

The phytochemical composition of CC, FCC, and the standard compound loganin was determined by HPLC. The same peaks were found between standard compound loganin and CC, FCC extracts. The total phenolic and flavonoid contents of CC and FCC extracts were measured according to a previously described method [26].

### 2.3. Animal Housing

Sprague-Dawley rats (220 ± 10 g, 7 weeks old) were acclimatized for 7 days according to animal welfare regulations of the Institutional Animal Care and Use Committee (IACUC; CBNU 2016-68) of Jeonbuk National University Laboratory Animal Center in South Korea. The temperature (25 ± 1 °C), humidity levels (50 ± 10%), and 12 h light/dark cycles were maintained during the experimental period [27]. 

### 2.4. Experimental Design

32 rats were randomly divided into 4 groups, *n* = 8: (1) control (saline administration alone), (2) stress (saline administration alone and immobilization stress), (3) CC 100 (CC 100 mg/kg administration and immobilization stress), and (4) FCC 100 (FCC 100 mg/kg administration and immobilization stress), groups. The saline or extract was orally administered to rats 1 h before immobilization. The dose of CC was orally administered to the rats was based on a previous study [28]. A 14-day stress-free toxicity test was performed prior to the formal study. CC or FCC (100 mg/kg) treatment did not show any signs of toxicity in rats, such as body weight, feed intake, and organ histological structure (data not shown). The volume of the gavage to the rats was 0.25 mL/100 g body weight. The rats were immobilized for 2 h per day for 14 consecutive days using a restraining chamber (15 × 5 × 5 cm). Depression-like behavioral testing was performed 24 h after the end of the immobilization stress. Rats were anesthetized with isoflurane (JW Pharmaceutical, Seoul, South Korea) after behavioral testing and then sacrificed. Blood was collected, and then centrifuged (3000× *g*, 4 °C, 15 min) after kept for 30 min at 22 °C. The hippocampus was removed on ice. The serum and hippocampus were stored at −80 °C until use.

### 2.5. Forced Swim Test (FST)

The FST is a common behavioral test used in the rodent model to determine the effectiveness of potential antidepressants. Rats were subjected to forced swim test (FST) on the 15th day after immobilization stress as described previously [29]. Each rat was forced to swim in an open glass cylinder (diameter 20 cm, height 50 cm) containing 30 cm of water (depth) at 25 ± 1 °C. At this depth, rats could not touch the bottom of the cylinder. On day 14, every rat was trained in the water-filled cylinder for 5 min. On day 15, rats were forced to swim for 5 min, and their climbing behavior was determined. Climbing behavior was defined as the upward-directed movements of the forepaws along the side of the swim chamber [29]. Immobility time was recorded during the 5 min test period in which the animal did not express escape responses. Each rat was deemed to be immobile when it stopped struggling and remained floating and motionless in the water, only performing necessary actions to keep its head above water. Rat behavior was recorded with a video camera throughout the testing period. The immobility time and climbing time of each rat were assessed by observers blinded. After the swimming test, rats were dried and put back in the cage. Water in the cylinder was changed for each test.

### 2.6. Oxidative Stress Markers Malondialdehyde (MDA) and Nitric Oxide (NO) Estimation in Hippocampus and Serum

MDA is generated by the degradation of polyunsaturated lipids, used as a biomarker for measuring the lipid peroxidation. The measurement of substance reactive with thiobarbituric acid is a well-established way to screen and monitor lipid peroxidation. Hippocampus and serum MDA were measured by thiobarbituric acid reactive substances (TBARS) kit (Cayman, Ann Arbor, MI, USA). Hippocampus and serum NO concentration was measured using the Griess Reagent kit (Thermo Scientific, Waltham, MA, USA) according to the manufacturers’ protocols.

### 2.7. Corticosterone, β-Endorphin, and Serotonin Evaluation

The concentrations of corticosterone, β-endorphin, and serotonin in the serum were assayed by corticosterone (R&D Systems, Minneapolis, MN, USA), β-endorphin, and serotonin (Elab Science, Wuhan, Hubei, China) ELISA kits. Detections were performed according to the manufacturers’ specifications.

### 2.8. Western Blot

Hippocampus and SH-SY5Y cells were lysed by tissue protein extraction reagent (T-PER) (Thermo Scientific, Waltham, MA, USA), or RIPA buffer (Biosesang, Gyeonggi-do, South Korea). After centrifugation (13000× *g*, 4 °C, 15 min), total protein concentration in the supernatant was measured with a bicinchoninic acid (BCA) protein assay kit (Thermo Scientific, Waltham, MA, USA). Protein was separated by 10–12% sodium dodecyl sulfate-polyacrylamide gel electrophoresis (SDS-PAGE) and transferred to a nitrocellulose membrane. The membrane was incubated with 5% non-fat milk (Bio-Rad Laboratories, California, USA) in Tris-buffered saline with Tween-20 (TBST) at 22 °C for 2 h. Primary antibodies were diluted according to the manufacturers’ specifications (Table 1) and incubated at 4 °C overnight. The membrane was washed with TBST and then incubated with secondary antibodies (goat anti-rabbit IgG-HRP) (Thermo Scientific, Waltham, MA, USA) for 2 h. Bands were detected using a clarity western chemiluminescent (ECL) substrate kit (Bio-Rad Laboratories, Hercules, California, USA), and band images were obtained using a LAS 500 image system (GE Healthcare, UK). The bands were quantified with Quantity One software (Bio-Rad Laboratories, Hercules, California, USA).

### 2.9. Cell Culture and MTT Assay

SH-SY5Y neuroblastoma cell line was obtained from Korean cell line bank (Seoul, South Korea) and grown in EMEM (ATCC, Manassas, Virginia, United States)/F12 medium (Gibco, Carlsbad, CA, USA) (1:1) supplemented with 10% FBS (GE Healthcare, Chicago, IL, USA) and 1% penicillin/streptomycin (Sigma-Aldrich, St. Louis, MO, USA) and cultured in 5% CO_2_ at 37 °C. Cell viability was measured using 3-(4,5-dimethylthiazol-2-yl)-2,5-diphenyltetrazolium bromide (MTT) (Sigma-Aldrich, St. Louis, MO, USA) assay [30]. SH-SY5Y cells were seeded in 96-well plates at a density of 1 × 10^4^ cells/well and cultured for 24 h. To assess the cytotoxicity, SH-SY5Y cells were treated with CC or FCC (20, 50, and 100 μg/mL) for 24 h. For cell viability, SH-SY5Y cells were pretreated with CC or FCC for 2 h and then co-incubated with 300 µM H_2_O_2_ for another 24 h. The 300 µM H_2_O_2_ was freshly prepared from a 30% (mass/mass) stock solution. The concentration and molar mass of the stock solution is 1.150 g/mL and 10.014 M. The stock solution was first diluted to 40 mM and then diluted to 300 µM in the medium. Morphologic images of SH-SY5Y cells were taken by an inverted microscope (Olympus, Tokyo, Japan) at fixed 400× magnification.

### 2.10. Estimation of ROS Generation in Cells

ROS generation was determined using the ROS kit (Sigma-Aldrich, St. Louis, MO, USA) according to the manufacturer’s instructions. Briefly, SH-SY5Y cells were seeded in 96-well black plates with clear bottoms at a density 1 × 10^5^ cells/mL. Cells were pretreated with CC or FCC for 2 h followed by co-culture with H_2_O_2_ for 3 h. The medium was removed and washed by Hanks’ Balanced Salt Solution (HBSS). The master reaction mix (100 µL) was added to the wells and then cells were incubated in 5% CO_2_, 37 °C incubator for 30 min. The fluorescence intensity was measured at an excitation of 640 nm and an emission of 675 nm.

### 2.11. LDH Release Detection in Cells

SH-SY5Y cells were dispensed into a 96-well plate at a density of 1 × 10^4^ cells/well. Cells were pretreated with CC or FCC (20, and 50 μg/mL) for 2 h followed by co-culture with H_2_O_2_ for 24 h. The release of lactate dehydrogenase (LDH) by the cells was determined using an LDH Cytotoxicity Detection assay kit (TAKARA, Shiga Prefecture, Japan) according to the manufacturer’s instructions.

### 2.12. RNA Extraction and Quantitative Real-Time PCR (qPCR)

Total RNA was extracted from cells according to the manufacturer’s instructions using a total RNA extraction kit (GeneAll, Seoul, South Korea). The cDNA was reverse-transcribed from 3 μg total RNA and the cDNA synthesis procedure was performed according to the manufacturer’s instructions (Thermo Fisher Scientific Korea Ltd. Seoul, South Korea). cDNA was subjected to quantitative real-time PCR (qPCR) on a CFX96 Real-Time PCR Detection System (Bio-Rad Laboratories, Hercules, CA) with SYBR Green I as double-stranded DNA-binding dye. After the reaction was finished, specificity was verified with the melting curve analysis. Quantification was performed by comparing Ct values of each sample normalized with glyceraldehyde-3-phosphate dehydrogenase (GAPDH) as an internal control. All PCR primers were obtained from Bioneer (Daejeon, South Korea) (Table 2).

### 2.13. Statistical Analysis

Data were expressed as the mean ± standard deviation (SD). Group comparisons were performed using one-way analysis of variance (ANOVA) and *t*-test using Prism 7.0 (GraphPad Software Inc., San Diego, CA, USA). A value of *p* < 0.05 was considered statistically significant.

## 3. Results

### 3.1. Evaluation of Active Compounds and the Contents in CC and FCC Extract

**Figure 1:**
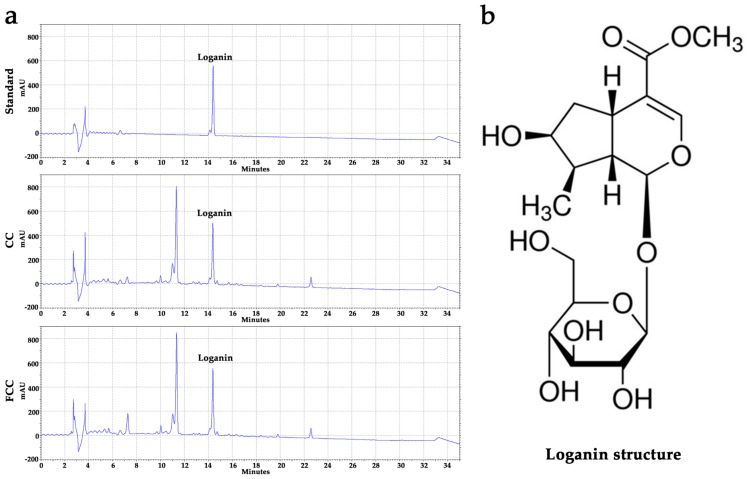
(**a**) HPLC profiles of the loganin standard for *Cornus officinalis* (CC) and fermented CC (FCC), (**b**) the structure of loganin.

Loganin was identified in water extracts of CC or FCC using HPLC analysis (Figure 1a). The peak preceding the loganin peak should be another compound. Based on the previous research and their HLPC analysis results, we suspected this compound is morroniside that contented in CC extract and will be investigated in the future. The loganin, total phenolic and flavonoid contents in CC and FCC are shown in Table 3. Loganin, phenolic, and flavonoid contents were significantly (*p* < 0.05) increased after fermentation.

### 3.2. CC and FCC Improved Depression-Like Behavior in Rats

**Figure 2:**
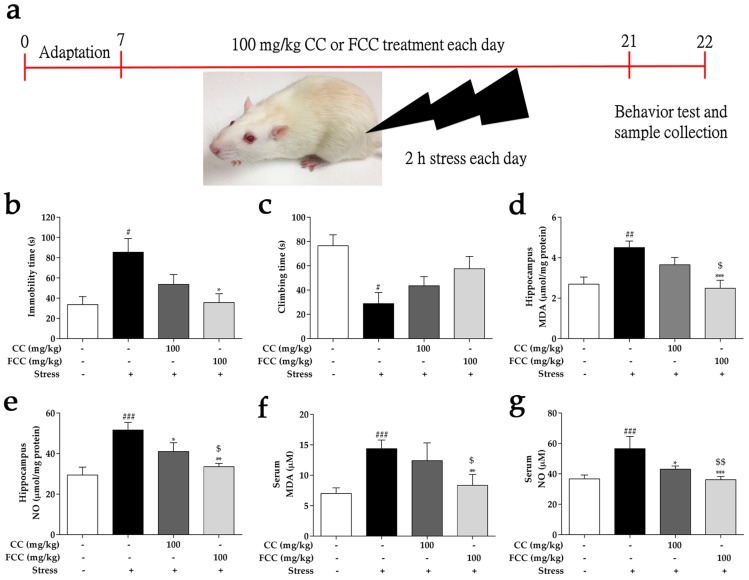
Pretreatment with CC or FCC relieved depression-like behavior and weakened oxidative stress in stress-induced rats. (**a**) Experimental design of rat immobilization stress. The (**b**) immobility time and (**c**) climbing time was measured in the forced swim test (FST). The oxidative stress markers (**d**,**f**) malondialdehyde (MDA) and (**e**,**g**) nitric oxide (NO) were estimated in rat hippocampus and serum by Thiobarbituric acid reactive substances (TBARS) and Griess reagent kits. “+, -”: Rats were treated with or without CC extract, FCC extract or stress. The data are expressed as mean ± SD, *n* = 8. *^#^ p* < 0.05, *^##^ p* < 0.01, *^###^ p* < 0.001 compared to the control group, * p < 0.05, ** p < 0.01, *** p < 0.001 compared to stress group, and ^$^ *p* < 0.05, ^$$^ *p* < 0.01 compared to CC 100 group.

Rats exposed to repeat immobilization stress for 14 days increased immobility time in the FST, suggesting stress-induced failure or behavioral despair compared to the control group. However, FCC (100 mg/kg) pretreatment significantly (*p* < 0.05) reduced immobility time in FST as compared with the stress group, suggesting that FCC administration decreased depressive-like behavior. In contrast, CC (100 mg/kg) pretreatment did not significantly decrease immobility time. The FCC pretreatment showed a much better ability to attenuate depression behavior (Figure 2b). “Climbing behavior” is another key method of behavior study [29]. In rats of the stress group, climbing behavior was significantly decreased (*p* < 0.05) during the FST as compared with control groups. However, experimental rats climbing behavior time was restored by pretreatment with 100 mg/kg CC or FCC (Figure 2c). FCC was comparatively better than CC at increasing climbing behavior time, despite weak statistical significance.

### 3.3. CC and FCC Attenuated Oxidative Stress in Rats

Oxidative stress is defined as the excessive production of free radicals that dramatically alter neuronal function. MDA and NO levels were markedly (*p* < 0.01 or *p* < 0.001) increased in the hippocampus and serum after rats were restricted for 2 h each day for 14 days. In addition, the CC and FCC pretreatment dramatically (*p* < 0.05, *p* < 0.01, or *p* < 0.001) reduced MDA and NO levels compared to the untreated stress group (Figure 2d–g). In particular, FCC significantly (*p* < 0.05 or *p* < 0.001) inhibited production of MDA and NO compared to CC.

### 3.4. CC and FCC Regulated Stress-Related Hormones in Stress-Induced Hippocampal Deficits in Rats

**Figure 3:**
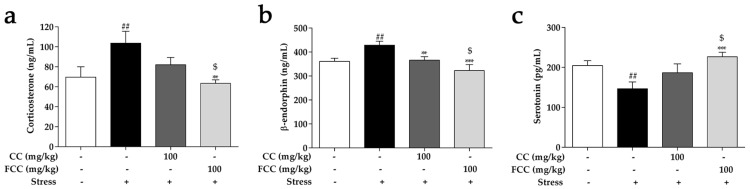
Pretreatment with CC or FCC regulated the stress-related hormones in stress-induced rats. Levels of (**a**) corticosterone, (**b**) β-endorphin, and (**c**) serotonin were evaluated by ELISA kits. “+, -”: Rats were treated with or without CC extract, FCC extract or stress. The data are expressed as mean ± SD, *n* = 8. *^##^ p* < 0.01 compared to the control group, ** *p* < 0.01, *** *p* < 0.001 compared to stress group, and ^$^ *p* < 0.05 compared to CC 100 group.

Levels of serum corticosterone and β-endorphin were notably (*p* < 0.01) increased in the stress group, while these level was dramatically (*p* < 0.01 or *p* < 0.001) reduced by pretreatment of CC or FCC extracts after immobilization stress (Figure 3a,b). Serum serotonin levels in stress group was significantly (*p* < 0.01) decreased compared to the normal control group, while FCC pretreatment effectively (*p* < 0.001) increased serotonin levels (Figure 3c). CC had no significant effect on the improvement of serotonin levels. Individually, FCC extract significantly (*p* < 0.05) regulated stress-related physiologic indicators compared to CC extract.

### 3.5. CC and FCC Blocked the MAPK/COX-2 Signaling Pathways in Rat Hippocampus

**Figure 4:**
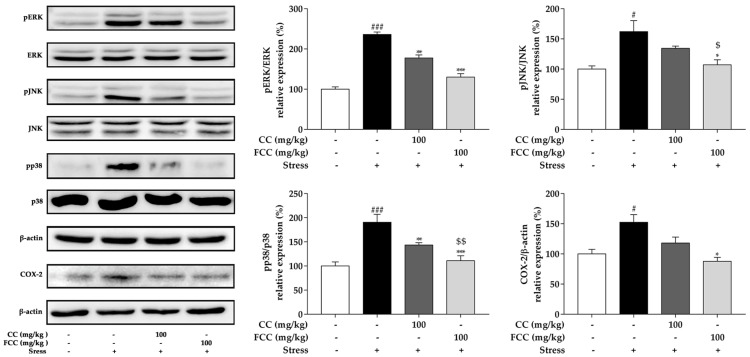
Neuroprotective of CC and FCC on stress-induced hippocampal deficits in rats by modulating mitogen-activated protein kinases (MAPK) (ERK, JNK, and p38) and COX-2 signaling pathway. The expressions of phosphorylation MAPK and COX-2 were evaluated by western blotting. “+, -”: Rats were treated with or without CC extract, FCC extract or stress. The data are expressed as mean ± SD, *n* = 8. *^#^ p* < 0.05, *^###^ p* < 0.001, compared to the control group and * *p* < 0.05, ** *p* < 0.01, *** *p* < 0.001 compared to stress alone group, and ^$^ *p* < 0.05, ^$$^ *p* < 0.01 compared to CC 100 group.

Previous study has shown that stress-induced oxidative stress may cause neurotoxicity by activating the mitogen-activated protein kinase (MAPK) family [25]. Our data showed that phosphorylation of MAPK (ERK1/2, JNK, and p38) family proteins was significantly (*p* < 0.05 or *p* < 0.001) increased in the hippocampus after stress challenge, but was remarkably (*p* < 0.05, *p* < 0.01 or *p* < 0.001) reversed by pretreatment with CC and especially FCC extract (Figure 4). FCC extract dramatically (*p* < 0.05 or *p* < 0.001) suppressed the MAPK (ERK1/2, JNK, and p38) family protein phosphorylation compared to CC extract. Stress induces oxidative stress and also causes inflammation. COX-2 is a key mediator of inflammatory pathways in rats [31], and increased COX-2 expression has been found in stressed rats [25]. CC or FCC pretreatment blocked increasing level of COX-2 expression and especially FCC pretreatment significantly (*p* < 0.05) reduced COX-2 expression level (Figure 4).

### 3.6. CC and FCC Alleviated H_2_O_2_-Induced Apoptosis in Cells

**Figure 5:**
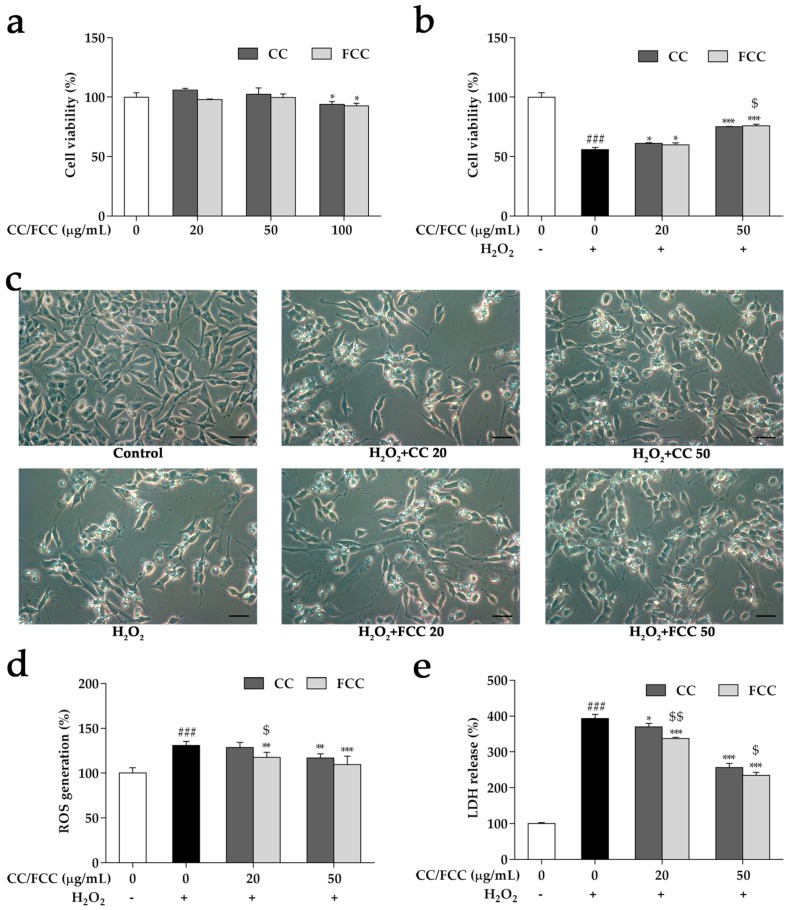
Effect of CC and FCC on morphology, cell viability and cytotoxicity in SH-SY5Y neuroblastoma cells. The (**a**) cell cytotoxicity and (**b**) cell viability were detected by 3-(4,5-dimethylthiazol-2-yl)-2,5-diphenyltetrazolium bromide (MTT). (**c**) Morphological changes (400×, scale bar: 20 µm) showed the cell viability was increased after CC or FCC pretreatment. (**d**) Reactive oxygen species (ROS) generation and (**e**) Lactate dehydrogenase (LDH) release caused by H_2_O_2_ was significantly decreased by pretreatment with CC or FCC. “+, -”: Cells were treated with or without H_2_O_2_. The data are expressed as mean ± SD, n = 3. *^###^ p* < 0.001 compared to the control cells and * *p* < 0.05, ** p < 0.01, *** p < 0.001 compared to H_2_O_2_ treatment alone, and ^$^ *p* < 0.05, ^$$^ *p* < 0.01 compared to CC group at the same dose.

SH-SY5Y were treated with CC or FCC at concentrations of 20, 50, and 100 µg/mL, and the toxic dose on cell viability was 100 µg/mL compared with untreated cells (*p* < 0.05) (Figure 5a). Neuroprotective effects of CC or FCC (20 and 50 µg/mL) were investigated in the H_2_O_2_-induced cell death in SH-SY5Y cells. Dose-dependent protection was observed by pretreatment with CC or FCC, and cell viability was dramatically (*p* < 0.05 or *p* < 0.001) increased compared with cells treated with H_2_O_2_ alone (Figure 5b). H_2_O_2_ induced changes the morphology of the cells but morphological changes were prevented by CC or FCC pretreatment in a dose-dependent manner (Figure 5c). In particular, FCC extract exhibited greater (*p* < 0.05) capability to increase cell survival than CC extract at the same dose.

### 3.7. CC and FCC Attenuated H_2_O_2_-Induced ROS Generation and LDH Release in Cells

H_2_O_2_ induces intracellular ROS production in SH-SY5Y neuroblastoma cells resulting in cell damage. Here, the effects of CC and FCC on H_2_O_2_-induced cytotoxicity was investigated by measuring the endogenous ROS generation and LDH release. The data showed that treatment with H_2_O_2_ led to a significant (*p* < 0.001) increase of intracellular ROS generation and LDH release; however, CC and FCC inhibited (*p* < 0.05, *p* < 0.01, or *p* < 0.001) ROS generation and LDH release caused by H_2_O_2_ in a dose-dependent manner (Figure 5d,e). Similarly, FCC exhibited greater (*p* < 0.05 or *p* < 0.01) inhibition of ROS and LDH generation relative to CC extract at the same dose. The results confirmed that CC and FCC had anti-oxidative effects on H_2_O_2_-mediated oxidative stress in SH-SY5Y cells.

### 3.8. CC and FCC Enhanced the Antioxidant Enzymes and Neuronal Stress Biomarkers in Cells

**Figure 6:**
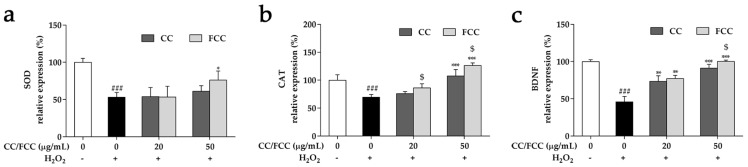
Effect of CC and FCC on antioxidant enzymes and neuronal stress marker in SH-SY5Y neuroblastoma cells. The antioxidant enzymes (**a**) superoxide dismutase (SOD), (**b**) catalase (CAT), and neuronal marker (**c**) brain-derived neurotrophic factor (BDNF) genes expression levels were detected by quantitative real-time PCR. “+, -”: Cells were treated with or without H_2_O_2_. The data are expressed as mean ± SD, *n* = 3. *^###^ p* < 0.001 compared to the control cells and * p < 0.05, ** p < 0.05, *** p < 0.001 compared to H_2_O_2_ treatment alone, and ^$^ *p* < 0.05 compared to CC 100 µg/mL treatment group.

The gene expressions of antioxidant enzymes superoxide dismutase (SOD), catalase (CAT), and neuronal stress biomarkers brain-derived neurotrophic factor (BDNF) were examined by quantitative real-time RT-PCR. The expressions of SOD and CAT were significantly (*p* < 0.001) decreased in SH-SY5Y cells treated with H_2_O_2_ (Figure 6a,b). Pretreatment with 50 µg/mL FCC notably (*p* < 0.05) reversed this decline. However, CC and 20 µg/mL FCC pretreatment showed a slight increase, despite weak statistical significance. The expression of BDNF was dramatically (*p* < 0.05) reduced after treated with H_2_O_2_ alone, whereas the decrease of BDNF was significantly (*p* < 0.01 or *p* < 0.001) increase after pretreatment with CC and especially FCC (*p* < 0.05) (Figure 6c). FCC had a robust capacity (*p* < 0.05) to control the levels of gene expression at the same dosage as CC extract.

### 3.9. CC and FCC Inhibited the H_2_O_2_-Induced Apoptosis in Cells

**Figure 7:**
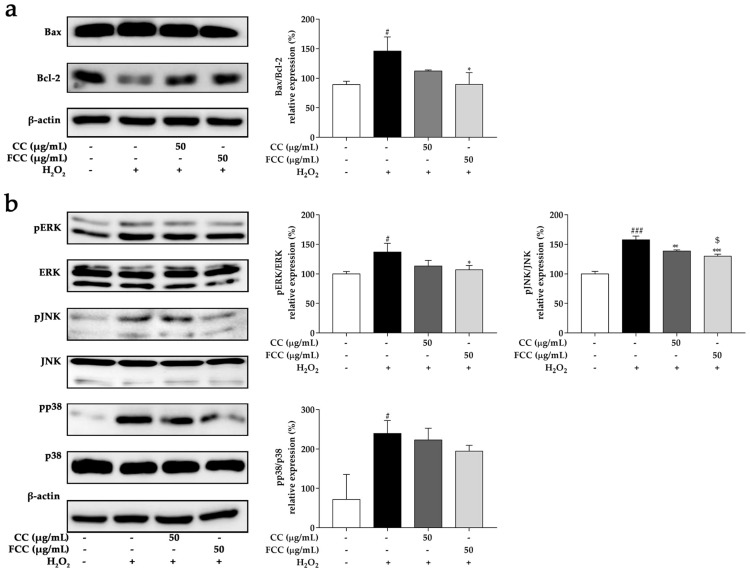
Effect of CC or FCC (50 µg/mL) on Bax and Bcl-2 family protein, and MAPK (ERK, JNK, and p38) protein expression in SH-SY5Y neuroblastoma cells. Cells were pretreated with CC or FCC (50 µg/mL) for 2 h, followed by co-treatment with H_2_O_2_ for another 24 h. (**a**) Bax and Bcl-2 family protein, and (**b**) phosphorylation MAPK protein expression levels were evaluated by western blotting. “+, -”: Cells were treated with or without CC, FCC or H_2_O_2_. The data are expressed as mean ± SD, *n* = 3. *^#^ p* < 0.05, *^###^ p* < 0.001, compared to the control group and * p < 0.05, ** p < 0.01, *** p < 0.001 compared to stress alone group, and ^$^ *p* < 0.05 compared to CC group at the same dose.

In order to further explore the neuroprotective role of CC and FCC on H_2_O_2_-treated SH-SY5Y cells, the protein expressions of pro-apoptotic factor Bax and anti-apoptotic factor Bcl-2 was examined by western blotting. The Bax/Bcl-2 ratio was drastically (*p* < 0.05) increased in SH-SY5Y cells treated with H_2_O_2_ alone but 50 µg/mL FCC markedly (*p* < 0.05) decreased the Bax/Bcl-2 ratio (Figure 7a). Thus, as compared with CC extract, FCC had greater regulation ability on Bax/Bcl-2 ratio.

### 3.10. CC and FCC Suppressed MAPK Signaling Pathways in Cells

Previous studies have shown that H_2_O_2_-induced oxidative stress may cause neurotoxicity by activating the MAPK family [32,33,34]. As shown in Figure 7b, the phosphorylation of MAPK family proteins was dramatically elevated in SH-SY5Y cells after were challenged with H_2_O_2_ alone. However, pretreatment with CC or FCC significantly decreased (*p* < 0.05, *p* < 0.01, or *p* < 0.001) phosphorylation of MAPK levels. In particular, FCC pretreatment displayed a better (*p* < 0.05) ability on inhibition phosphorylated JNK expression than CC pretreatment. These results indicated that CC and FCC protected cells from oxidative stress by inhibiting the activation of MAPK pathway members.

## 4. Discussion

In this study, we investigated the neuroprotective effects of CC and FCC using stress-induced hippocampal deficits in rats and H_2_O_2_-induced apoptosis in SH-SY5Y cells. The present study showed that immobilization stress caused oxidative stress in rats. MDA and NO were significantly increased in stressed rats, which is consistent with the previous reports [35,36]. Pretreatment with CC and FCC reduced the both MDA and NO production in rats subjected to immobilization stress. CC and FCC extract may effectively scavenge free radicals and prevent lipid peroxidation, thereby reducing stress-induced brain damage and achieving anti-depressant properties. 

Corticosterone is a marker of susceptibility to oxidative/nitrosative cerebral damage following exposure to stress in rats [37] and is the main glucocorticoid in rodents. The hippocampus contains high proportions of glucocorticoid receptors [38]. High glucocorticoid levels trigger ROS to cause oxidative damage in the hippocampus, resulting in cognitive impairment [39]. Serum corticosterone level was measured in rats immediately after stress. The present data show that the corticosterone levels in the stress group was increased, while pretreatment with CC and FCC extract reversed the increase in serum corticosterone. It seems that CC and FCC could protect the hippocampus from stress-induced damage by regulating the levels of corticosterone. Kalynchuk et al. proved that the duration of immobility in the FST increased due to dose-dependent concentration of corticosterone [40]. In the present study, the immobility time also increased after the rats immobilized for 14 days, but reversed by CC and FCC pretreatment. Behavioral tests demonstrated that pretreatment with CC and FCC attenuated depression-like behavior in rats. β-endorphin is an endogenous opioid that acts as a stress-relieving factor [41]. The opioid system plays an important role in regulating the pro-oxidative and anti-oxidative balance [42]. In this study, serum β-endorphin levels was significantly increased after immobilization stress. However, pretreatment with CC and FCC extract significantly decreased the levels of β-endorphin compared to the immobilization stress group. These results showed that CC and FCC had anti-stress effects and decreased the stress-induced oxidative stress.

Serotonin is produced by nerve cells and transmits signals between nerve cells and plays a crucial role in the response to stress and antidepressant action [43]. Abnormal serotonin levels causes depression and anxiety disorders. Furthermore, p38 MAPK activation has direct impacts on the serotonin system and elevates serotonin transport (SERT) activity via a trafficking-independent, protein phosphatase 2a-dependent process [44]. In the present study, rats challenged by stress exhibited higher activation of p38 MAPK which can elevate SERT activity, resulting the reduction in peripheral 5-HT. Perhaps this is why we found that the serum levels of serotonin was decreased in stressed rats, although the connection between peripheral serotonin and central serotonin signaling centrally is tenuous at best. Pretreatment with CC and FCC significantly decreased the p38 MAPK phosphorylation and increased the serum serotonin concentration. Further research is needed to determine whether CC and FCC directly increase serotonin levels. In turn, the FCC can better regulate hormone levels by antagonizing stress and decreasing oxidative stress.

The H_2_O_2_-induced cell death model in SH-SY5Y cells is commonly used to investigate neuronal death induced by oxidative stress and the neuroprotective properties of natural products [45,46]. From our data, it was obvious that the H_2_O_2_-induced cell death was significantly attenuated by CC or FCC pretreatment. The protective effects of CC and FCC were further confirmed by the LDH assay. CC or FCC pretreatment significantly suppressed LDH activity, indicating a cytoprotective effect against H_2_O_2_-induced oxidative stress. The protective effect exerted by CC and FCC on H_2_O_2_-induced cytotoxicity was further supported by the MTT assay and the morphological observation.

H_2_O_2_ is the main ROS related to oxidative stress. It easily penetrates into cells and generates highly reactive hydroxyl radicals, thus damaging cellular components like including lipid, protein and DNA. H_2_O_2_ increases oxidative stress damage by increasing ROS generation [47], which leads to an imbalance of cellular oxidants and antioxidants. SOD and CAT enzymes are two active scavengers of superoxide and hydrogen peroxide. However, elevated ROS production or poor antioxidant defense mechanism can cause progressive cell damage, physiological dysfunction, and disease [48]. In the present study, the expressions of SOD and CAT genes were reduced in SH-SY5Y cells after treated with H_2_O_2_. However, the low expression of these genes caused by H_2_O_2_ was attenuated by pretreatment with CC and FCC. The expression of SOD and CAT genes significantly increased in a dose-dependent manner. The decrement of ROS may be associated with increased expression and activities of SOD, CAT. Previous studies showed that the neuroprotective effects were due to reduced ROS production and the protein expression of the antioxidant enzymes SOD and CAT [49,50].

BDNF plays a major role in neural development and plasticity in both health and disease [51], and it is active in the hippocampus, cortex and basal forebrain which are vital to learning, memory and higher thinking. Meanwhile, the expression of BDNF plays a key role in the survival and differentiation of dopaminergic neurons. In the present study, cells exposed to H_2_O_2_ showed a low expression level of BDNF, while CC and FCC prevented a decrease in expression of BDNF. Thus, elevated the expression of BNDF by CC and FCC, which may increase the survival rate of the human dopaminergic cells, thus achieve neuroprotective effects against H_2_O_2_-induced oxidative stress [52].

The Bcl-2 family is well characterized as involved in the apoptotic process. Bax and Bcl-2 proteins control the permeabilization of the mitochondrial membrane and the release of apoptotic factors from the intermembrane mitochondrial space. The Bax/Bcl-2 ratio was significantly increased in this study after being treated with H_2_O_2_, whereas pretreatment with CC or FCC suppressed the Bax/Bcl-2 ratio increase in SH-SY5Y cells. Since, the Bax/Bcl-2 ratio is considered to be a valuable predictor of apoptotic cell death. These findings suggest that CC and especially FCC exist the ability to balance Bax and Bcl-2-dependent apoptotic pathways. Excessive oxidative stress produces oxidative free radicals such as ROS, which stimulate MAPK cascade phosphorylation [53]. MAPKs are serine-threonine protein kinases that play a key role in signal transduction. MAPK pathways are influenced by both receptor-ligand interactions and various stressors placed on cells. Stress or H_2_O_2_ were observed to activate MAPKs; however, CC and particularly FCC effectively inhibited MAPK family phosphorylation in both rats and cells, except that p38 MAPK in SH-SY5Y cells was not significantly reduced. Furthermore, COX-2 triggers pro-inflammatory processes that exacerbates neuronal degeneration and dysfunction in many neurological diseases [54]. We found a significant increase in COX-2 expression in the stressed rat hippocampus. However, pretreatment with CC and especially FCC gradually attenuated this effect. Taken together, CC and especially FCC showed neuroprotective properties through blocking MAPK signaling pathway.

## 5. Conclusions

**Figure 8:**
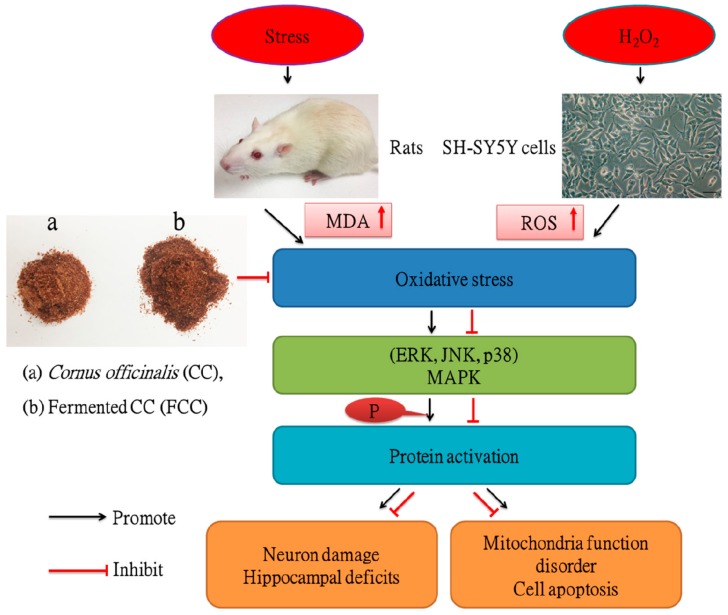
A schematic diagram illustrating the protective mechanism of *Cornus officinalis* (CC) and fermented CC extract against oxidative stress. CC and FCC extracts decreased the MDA and ROS levels in rats and cells, suppressed the MAPK phosphorylation to inhibit stress-induced hippocampal deficits and H_2_O_2_-induced oxidative stress.

The present data demonstrated that CC and especially FCC may mitigate the damage impact on the brain and inhibit H_2_O_2_-induced neurotoxicity in SH-SY5Y Cells by reducing oxidative stress (The summarized mechanistic pathways are shown in Figure 8). Therefore, these proposed that CC and FCC could be used as a natural auxiliary phytomedicine to prevent oxidative stress-induced neurodegenerative diseases.

## Figures and Tables

**Table 1:** Primary antibodies information.

Antibody	Host	Manufacturer	Cat. No.	Dilute Ratio
Bcl-2	Rabbit	Cell Signaling, USA	3498	1:1000
Bax	Rabbit	Santa Cruz Biotechnology, USA	sc-493	1:500
COX-2	Rabbit	Cell Signaling, USA	12282	1:1000
p-JNK	Rabbit	Cell Signaling, USA	9251	1:1000
JNK	Rabbit	Cell Signaling, USA	9252	1:1000
p-ERK	Rabbit	Cell Signaling, USA	9101	1:1000
ERK	Rabbit	Cell Signaling, USA	9102	1:1000
p-p38	Rabbit	Cell Signaling, USA	9211	1:1000
p38	Rabbit	Cell Signaling, USA	9212	1:1000
β-actin	Rabbit	Cell Signaling, USA	4970	1:1000

**Table 2:** Sequences of primers for use in the experiment.

Gene Target	Primer Name	Primer Sequence (5′-3′)	GenBank Accession No.
Catalase	CAT F	CCTTTCTGTTGAAGATGCGGCG	NM_001752
	CAT R	GGCGGTGAGTGTCAGGATAG	
Superoxide dismutase	SOD F	AGGCCGTGTGCGTGCTGAAG	NM_000454
	SOD R	CACCTTTGCCCAAGTCATCTGC	
BDNF	BDNF F	ATGACCATCCTTTTCCTTACT	NM_170731
	BDNF R	GCCACCTTGTCCTCGGAT	
GAPDH	GAPDH F	TTCACCACCATGGAGAAGGC	NM_001357943
	GAPDH R	GGCATGGACTGTGGTCATGA	

**Table 3:** Total phenolic and flavonoid contents of CC and FCC. All data were expressed as the mean ± SD, *n* = 3. * p < 0.05 compared to CC extract.

Sample	Logainin (mg/g)	Total Phenolic (mg GAE/g)	Total Flavonoid (mg RU/g)
CC	9.05 ± 0.05	112.95 ± 1.94	12.45 ± 0.33
FCC	9.83 ± 0.04 *	120.98 ± 0.16 *	15.79 ± 0.15 *

Note: Gallic acid and rutin were used as standards. Total phenolic and flavonoid contents were expressed in milligrams of gallic acid equivalent per gram of extract sample (mg GAE/g) and milligrams of rutin equivalent per gram of extract sample (mg RU/g).
